# Construction and Evaluation of Recombinant Pseudorabies Virus Expressing African Swine Fever Virus Antigen Genes

**DOI:** 10.3389/fvets.2022.832255

**Published:** 2022-04-13

**Authors:** Liyi Chen, Xinheng Zhang, Guanming Shao, Yangyang Shao, Zezhong Hu, Keyu Feng, Zi Xie, Hongxin Li, Weiguo Chen, Wencheng Lin, Hengxing Yuan, Hailong Wang, Jun Fu, Qingmei Xie

**Affiliations:** ^1^Heyuan Branch, Guangdong Provincial Laboratory of Lingnan Modern Agricultural Science and Technology and Guangdong Provincial Key Lab of Agro-Animal Genomics and Molecular Breeding, College of Animal Science, South China Agricultural University, Guangzhou, China; ^2^Guangdong Engineering Research Center for Vector Vaccine of Animal Virus, Guangzhou, China; ^3^Key Laboratory of Animal Health Aquaculture and Environmental Control, College of Animal Science, South China Agricultural University, Guangzhou, China; ^4^State Key Laboratory of Microbial Technology, Helmholtz International Lab for Anti-infectives, Institute of Microbial Technology, Shandong University–Helmholtz Institute of Biotechnology, Shandong University, Qingdao, China

**Keywords:** African swine fever virus, recombinant, pseudorabies virus, Red/ET recombineering technology, vaccine

## Abstract

African swine fever (ASF) is a highly contact infectious disease caused by the African swine fever virus (ASFV). The extremely complex structure and infection mechanism make it difficult to control the spread of ASFV and develop the vaccine. The ASFV genome is huge with many antigenic genes. Among them, *CP204L* (p30), *CP530R* (pp62), *E183L* (p54), *B646L* (p72), and *EP402R* (CD2v) are involved in the process of the virus cycle, with strong immunogenicity and the ability to induce the body to produce neutralizing antibodies. In this study, the recombinant virus rBartha-K61-pASFV that expresses the above ASFV antigen genes was constructed by Red/ET recombineering technology using pseudorabies virus (PRV) vaccine strain Bartha-K61. Western blot analysis showed that the ASFV antigen gene was expressed and the recombinant virus showed good genetic stability and proliferation characteristics in 15 continuous generations on porcine kidney (PK15) cells. The results of immunoassay of piglets and mice showed that rBartha-K61-pASFV had good immunogenicity and could induce higher antibody levels in the body. Therefore, PRV was a promising viral vector for expressing the ASFV antigen gene, and all the experiments in this study laid a foundation for the further development of a new viral vector vaccine of ASFV.

## Introduction

African Swine Fever (ASF), caused by the African swine fever virus (ASFV), is a highly contagious disease, leading to acute deaths in domestic pigs and wild boars with a mortality rate of nearly 100%. The pigs affected with ASFV will present typical ASF hemorrhagic lesions (1), such as petechial hemorrhages in the endocardium, hyperemic splenomegaly, hepatic congestion and hepatomegaly, alveolar edema, and systemic lymphadenitis. AFS was firstly observed in Kenya and once spread to most sub-Saharan African countries, the Caribbean, the EU Baltic States, and Poland (2). In August 2018, the first outbreak of ASF was occurred in Liaoning, China and quickly spread to 12 other provinces, killing 100,000 pigs and causing cumulative economic losses of over $20 million (3).

The African swine fever virus is a double-stranded DNA virus with icosahedral morphology (4). Its genome encodes more than 150 proteins, such as certain viral enzymes, proteins involved in viral transcription and replication, proteins involved in viral assembly, structural proteins, and others (5). The CD2v protein, a host immunoregulatory protein, is encoded by the *EP402R* gene of ASFV, existing in the capsule of virions with strong immunogenicity. In addition, ASFV knockout of the *EP402R* gene significantly reduces viremia and viral transmission, so the *EP402R* gene is an important candidate target for the development of vaccines (6). Polyprotein pp62 is a structural protein that is encoded by the *CP530R* gene, which exists in the core-shell of virus particles and can be hydrolyzed by a protease to the two core proteins of ASFV: p35 and p15 (7). Polyprotein pp62 is necessary for the correct assembly and maturation of the core of the viral particle. Its repression leads to an increase in the number of immature-like particles and to the accumulation of defective particles (8). ASFV p72, p30, and p54 are three of the most antigenic viral proteins, respectively, encoded by virus genes *B646L, CP204L*, and *E183L* (9). The expression of p30 is generally observed from 2 to 4 h post-infection (10). p30 has strong immunogenicity and induces the body to produce neutralizing antibodies, which is often used as an early detection protein for ASFV infection. p72 and p54 are generally expressed late in viral infection and are critical for viral replication. p72 protein has strong antigenicity and little difference in amino acid sequence between different strains. Compared with other structural proteins of ASFV, p72 has better stability, so it can be used as the main indicator for detecting ASFV infection (11).

Pseudorabies disease is an acute infectious disease that is caused by the pseudorabies virus (PRV), which can cause reproductive dysfunction in pigs. PRV is a member of the *Alphaherpesvirinae* subfamily of the *Herpesviridae* family (12). It is a double-stranded linear DNA virus with a 150 kb genome (13). In addition, the PRV genome contains several non-essential genes, multiple exogenous genes can be inserted and stably expressed (14). Studies have shown that non-essential region genes, such as TK, PK, gE, gI, and gG, can be used as the insertion site of a foreign gene. The deletion mutation of gD, gE, gI, and TK can make the wild strain PRV invade the central nervous system (15) and significantly reduce the virulence of the virus. Therefore, PRV can be used as an ideal vector of the viral vector vaccine. In addition, the Bartha-K61 strain is a well-known modified live vaccine, which has played a key role in global control and eradication of PR and has been widely used in China since the 1980s (16). Bartha-K61 strain was also successfully used as vectors for the expression of antigens of other swine pathogens. For example, a PRV recombinant expressing envelope glycoprotein E1 of hog cholera virus (17) and a hemagglutinin (HA)-expressing PRV recombinant of vaccine strain Bartha (18), both developed high levels of neutralizing antibodies and showed good safety and immunogenicity.

Recombinant viral vector vaccine is a new type of vaccine, which modifies specific virus genome by DNA recombination technology and expresses exogenous antigen genes into the vector virus (19, 20). Red/ET recombineering technology is an accurate DNA modification technique using homologous recombination-mediated by recombinase Redα/β or RecE/T (21). Compared with the traditional methods, this technique has the advantages of being free from the restriction of the enzyme restriction site, gene size restriction, no trace modification, and fast operation (22). In this study, Red/ET recombineering technology was used to, respectively, insert ASFV *CP204L* (p30), *CP530R* (pp62), *E183L* (p54), *B646L* (p72), and *EP402R* (CD2v) into the TK site of PRV vaccine strain Bartha-K61 and construct recombinant PRV expressing *CP204L, CP530R, E183L, B646L*, and *EP402R* genes of ASFV. This study laid a foundation for the development of a new viral vector vaccine for ASFV.

## Materials and Methods

### Viruses, Cells, and Animals

Vero and porcine kidney (PK15) cells were cultured at 37°C and 5% CO_2_ in complete Dulbecco's modified Eagle medium (DMEM) (C11995500BT, Thermo Fisher) supplemented with 10% fetal bovine serum (FBS) (10,099-141, Gibco). The PRV vaccine strains, Bartha-K61, were purchased from Qilu Animal Health Products (Shandong, China), and PRV (rBartha-K61) was improved by Helmholtz International Lab for anti-infectives of Shandong University (Shandong, China). Twenty-one-day-old BALB/c mice were provided by the Laboratory Animal Center of Southern Medical University. The experiment of a piglet was conducted on a pig farm in Shandong, China.

### Plasmids and Virus Construction

The plasmid pUC57-CMV-pASFV containing *CP204L* (NCBI Reference Sequence: QBH90581.1), *CP530R* (NCBI Reference Sequence: QBH90582.1), *E183L* (NCBI Reference Sequence: QBH90613.1), *B646L* (NCBI Reference Sequence: QBH90570.1), and *EP402R* (NCBI Reference Sequence: QBH90546.1) gene fragment was synthesized by Sangon Biotech (Shanghai, China). The ASFV antigen gene pASFV containing the homologous arm of the TK site was amplified by PCR. The recombinant plasmid pBeloBAC11-Bartha-K61-ΔTK-pASFV was constructed by Red/ET recombineering technology and verified to have no mutation by sequencing.

To rescue recombinant PRV (PRV-ΔTK-pASFV), 5 μg of recombinant plasmid was transfected into Vero cells by using Lipofectamine^®^ 3000 (L3000015, Invitrogen). The virus culture was harvested when the cytopathic effect (CPE) reached 90% or more. Finally, the recombinant virus PRV-ΔTK-pASFV was amplified and purified in Vero cells by four rounds of phagocytosis.

### Western Blotting

Porcine kidney cells were infected with rBartha-K61-pASFV at a multiplicity of infection (MOI) of 1. At 48 h post-infection, cell proteins were separated by sodium dodecyl sulfate-polyacrylamide gel electrophoresis (SDS-PAGE) and transferred to the PVDF membrane. The membranes were incubated with antibodies against *CP204L, CP530R, E183L, B646L*, and *EP402R* (1:1,000 dilution, produced by Sangon Biotech). Horseradish peroxidase (HRP)-conjugated goat anti-mouse immunoglobulin G (IgG) or goat anti-rabbit IgG (Proteintech Group, Inc., USA) was used as the secondary antibody as appropriate. Protein bands were imaged using an Azure c300 digital imager system (Azure Biosystems, Dublin, CA, USA).

### Growth Properties *in vitro*

Growth properties *in vitro* were evaluated with PK15 cells that were infected with rBartha-K61-pASFV at an MOI of 1. At 6, 12, 24, 36, 48, 60, 72, and 96 h post-infection, the supernatants were collected, and the 50% tissue culture infective dose (TCID_50_) was calculated by the Reed-Muench method.

### Animals' Immunization

Twenty-one-day-old BALB/c mice were subcutaneously injected with the same amount of rBartha-K61, rBartha-K61-EGFP (TK), rBartha-K61-pASFV (containing rBartha-K61-*CP204L*, rBartha-K61-*CP530R*, rBartha-K61-*E183L*, rBartha-K61-*B646L*, rBartha-K61-*EP402R*), and DMEM, and the inoculation doses was 10, and DMEM, and the inoculation dose was 10^5.0^TCID_50_. Piglets free of PRV antibody were intramuscularly injected with the same amount of rBartha-K61, rBartha-K61-EGFP (TK), rBartha-K61-pASFV, and DMEM, the inoculation dose was 10^5.0^TCID_50_, and strengthened by the second inoculation 2 weeks later. The p72 antibody levels were detected by the ASFV ELISA Kit (Qiantu Bio, Shanghai, China).

### Statistical Analysis

All experiments were repeated at least 3 times independently. The unpaired *t*-test of GraphPad Prism 8.0 was used to analyze data differences between groups, and *p* < 0.05 was considered statistically significant. Data are presented as the mean ± SD in the same treatment.

## Results

### Construction of the Recombinant Virus PRV-ΔTK-pASFV

**Figure 1:**
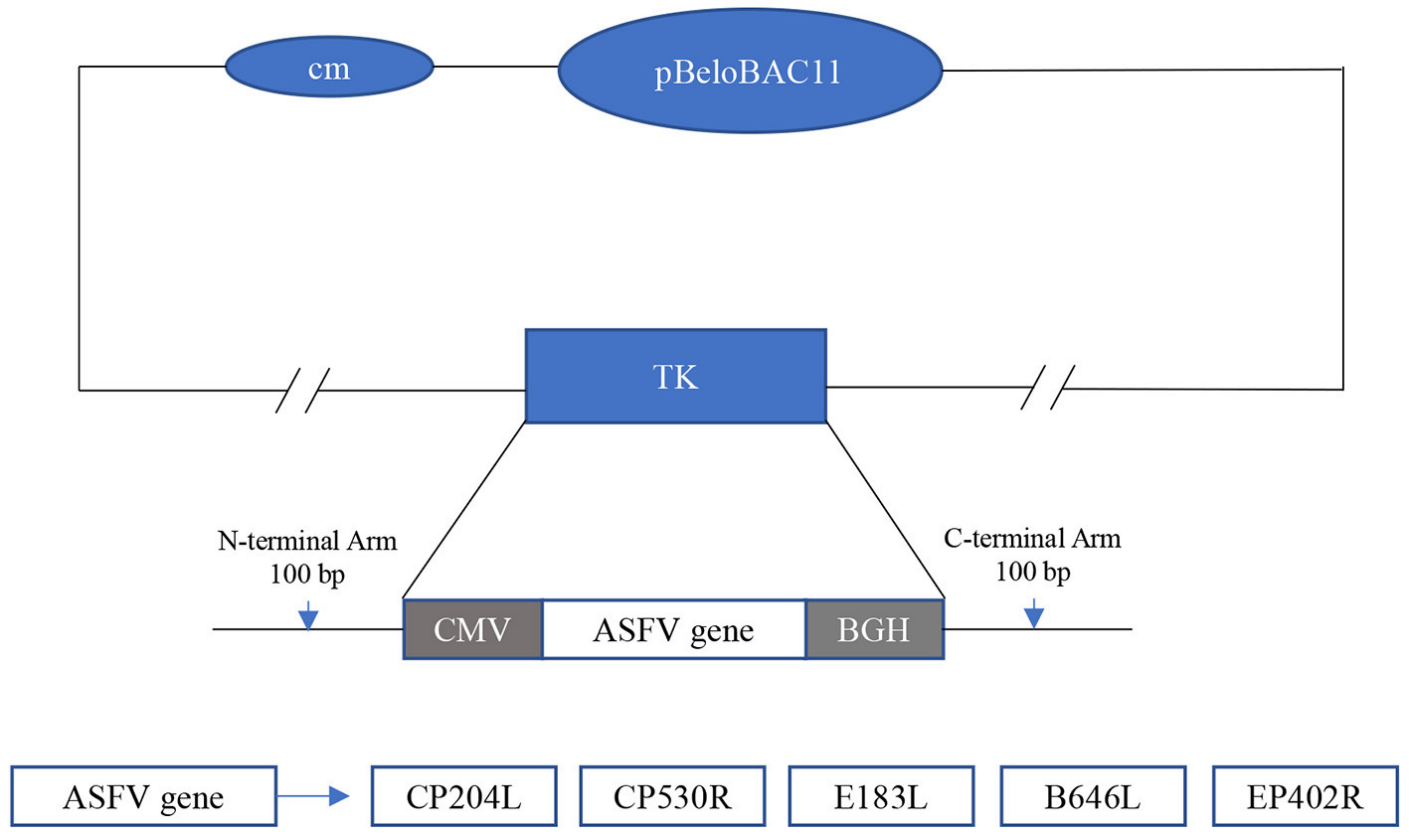
Construction of the recombinant virus PRV-ΔTK-pASFV. The vaccine vector used is an improved PRV vector. The gene of ASFV was inserted at the TK and was driven by the CMV promoter. The inserted ASF antigen genes included *CP204L, CP530R, E183L, B646L*, and *EP402R*.

As shown in Figure 1, the recombinant PRV PRV-ΔTK-pASFV expressing ASFV protein is constructed by homologous recombination using the Red/ET recombineering technology. The recombinant fragment of the ASFV antigen gene was inserted into the TK site to construct PRV-ΔTK-pASFV. The recombinant virus was transmitted continuously to 20 generations on PK15 cells, and the sequencing results showed that the exogenous genes were genetic stably without deletion and mutation.

### Identification of Recombinant Virus

**Figure 2:**
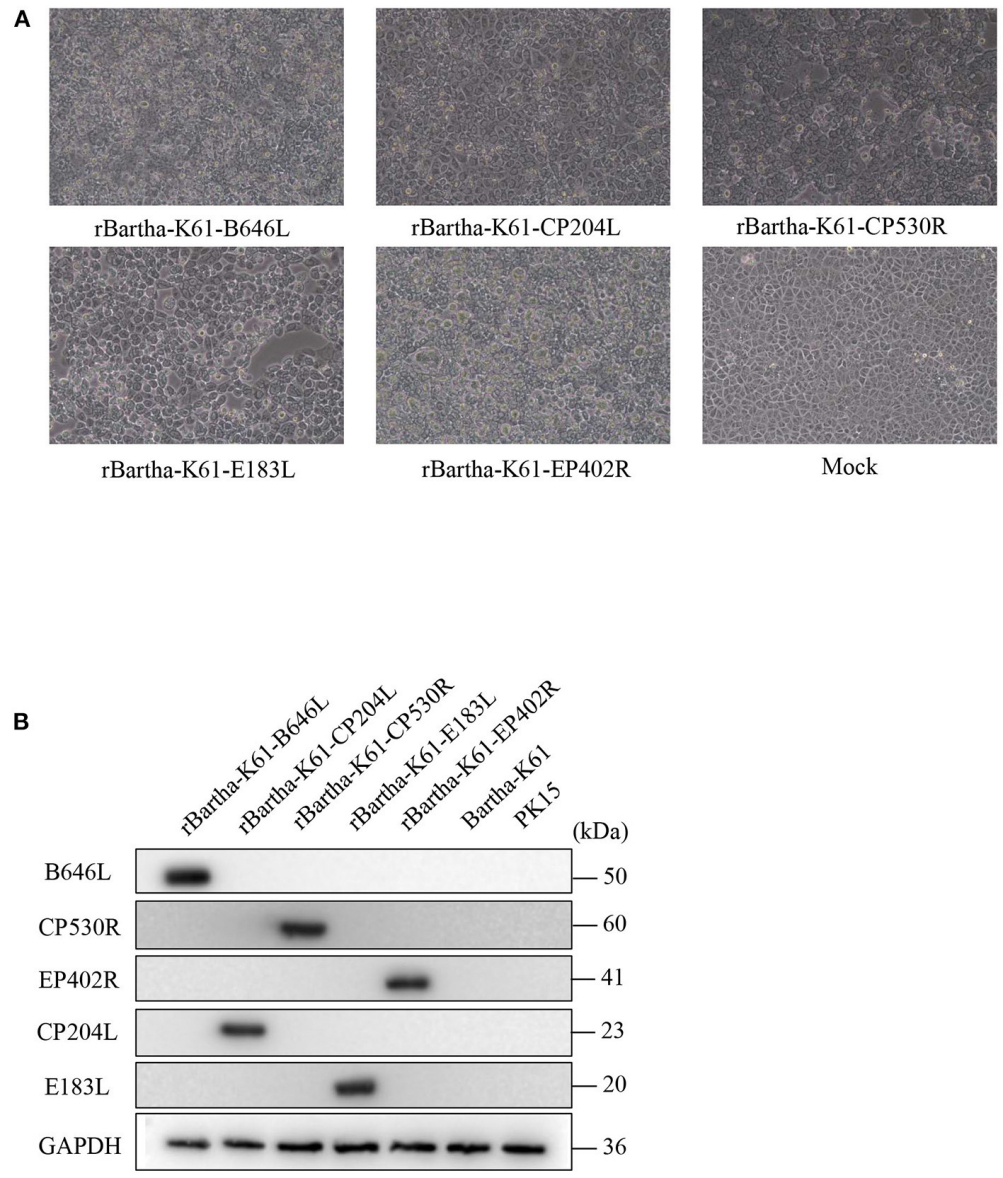
Virus rescue and identification of recombinant plasmid. **(A)** Cytopathic analyses of rBartha-K61 and Bartha-K61 in Vero cells. Vero cells were infected with each virus at an MOI of 1. At 48 h post-infection, cytopathic effects were observed (10×). **(B)** Porcine kidney (PK15) cells were challenged with rBartha-K61-pASFV at an MOI of 1. At 48 h post-infection, cell lysates were collected and analyzed by western blotting. Western blotting was detected with antibodies against the B646L, CP530R, ep402R, CP204L, and E183L proteins. The results are representative of the 3 independent experiments. GAPDH was used as sample-loading control.

The plasmid pBeloBAC11-Bartha-K61 was transfected into Vero cells for 48 h. The supernatant was harvested and inoculated into PK15 cells for blind transmission for 5 generations. At the fifth generation of passage, rBartha-K61 F5 was harvested and identified. The PK15 cells infected with rBartha-K61 showed enlargement and were rounded, followed by degeneration and rounded shrinkage. Finally, the PK15 cells were exfoliated and aggregated to form typical CPE, such as plaques (Figure 2A).

To verify the exogenous protein expression of the recombinant virus, rBartha-K61-pASFV was inoculated into PK15 cells at MOI = 1 for Western blot. As shown in Figure 2B, the ASFV proteins (*CP204L, CP530R, E183L, B646L*, and *EP402R*) are expressed in recombinant virus and the group Bartha-K61 or PK15 cell is not. The results showed that the ASFV protein could be stably inherited and expressed in the recombinant virus after multiple passages in cells.

### Growth Properties of Recombinant PRV Strains

**Figure 3:**
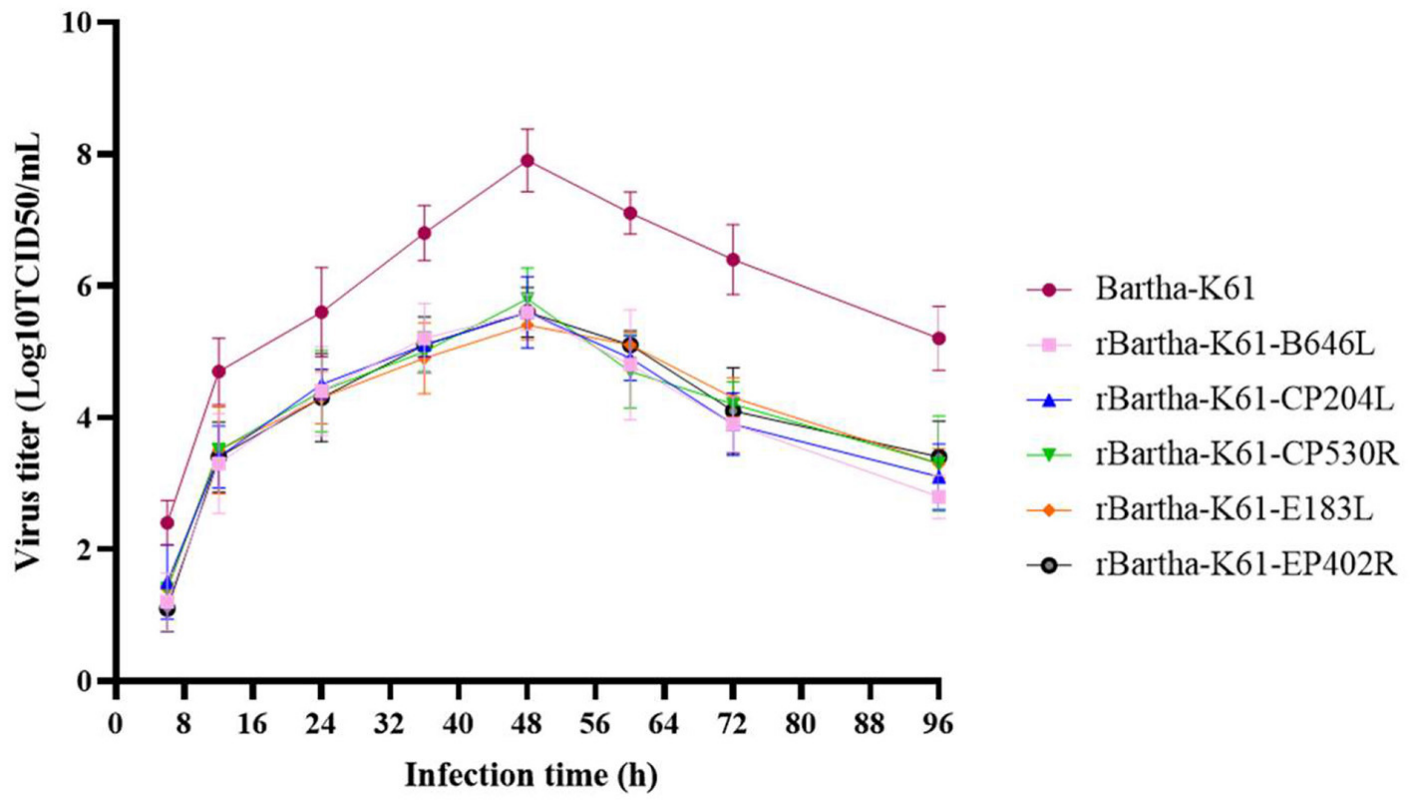
Growth properties of recombinant PRV strains. *In vitro* growth properties of parental (Bartha-K61) and recombinant viruses in PK15 cells following infection at an MOI of 1. The viral titers were determined in PK15 cells. At 6, 12, 24, 36, 48, 60, 72, 96 h post infection, the supernatants were collected, and the 50% tissue culture infective dose (TCID_50_) was calculated by the Reed-Muench method. Bars represent the mean ± SD of three independent experiments (*n* = 3).

To compare the growth properties of parental (Bartha-K61) and recombinant viruses, PK15 cells were inoculated with the viruses at an MOI of 1. Cellular supernatants were collected at different time points for virus titration. As shown in Figure 3, the recombinant virus could proliferate rapidly on the susceptible cell PK15, and the peak value of the virus titer is 1 × 10^5.8^ TCID_50_/ml. The growth properties of the recombinant virus were less capable of its parent strain Bartha-K61, but the proliferation trend was the same, suggesting that the inserted genes do not change the virus growth properties *in vitro*.

### Safety Evaluation of Recombinant Virus in Mice and Susceptible Piglets

**Figure 4:**
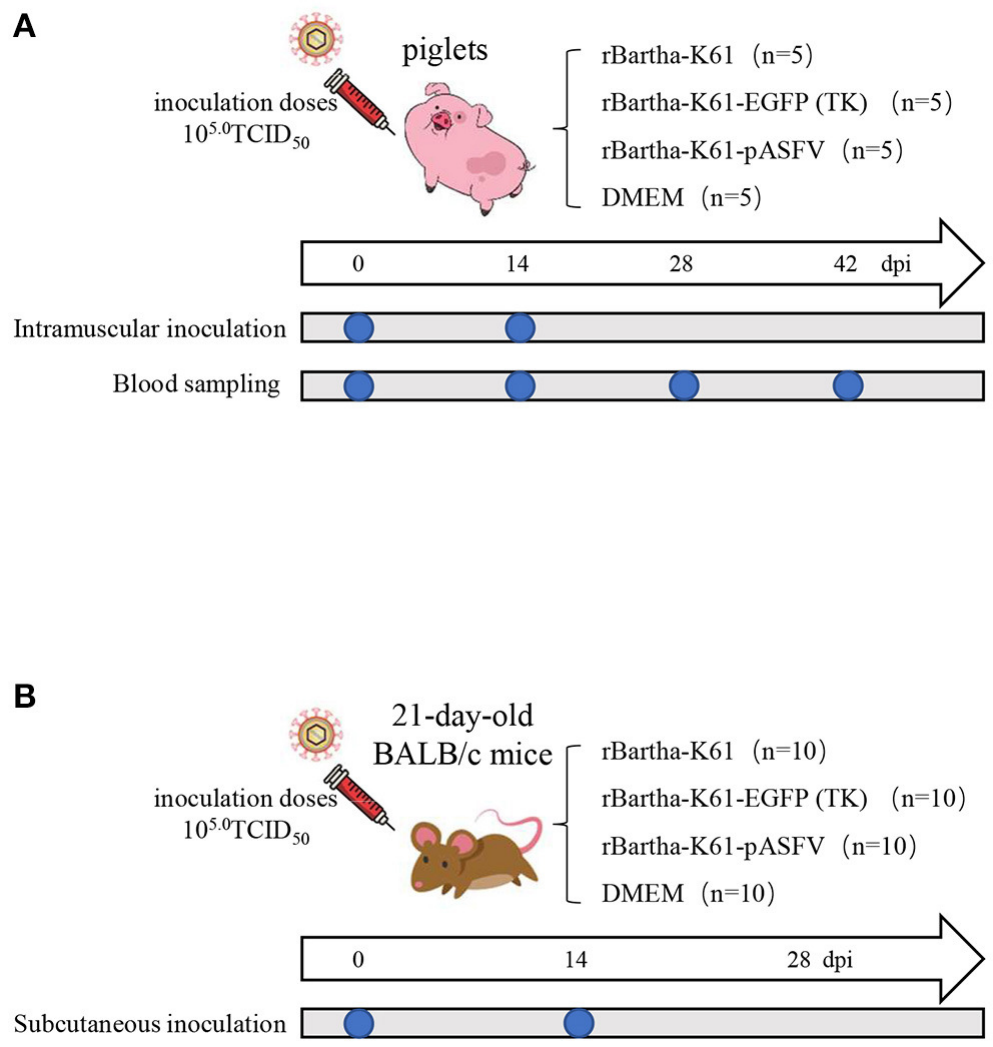
Animals' immunization and sample collection. Twenty-one-day-old BALB/c mice and piglets were subcutaneously injected with the same amount of rBartha-K61, rBartha-K61-EGFP (TK), rBartha-K61-pASFV (containing rBartha-K61-*CP204L*, rBartha-K61-*CP530R*, rBartha-K61-*E183L*, rBartha-K61-*B646L*, rBartha-K61-*EP402R* and DMEM, and the inoculation doses was 10^5.0^TCID_50_, which were strengthened by the second inoculation two weeks later (14 dpi). Furthermore, blood samples of piglets were collected at 0 dpi, 14 dpi, 28 dpi, and 42 dpi, to separate serum and detect antibody.

**Figure 5:**
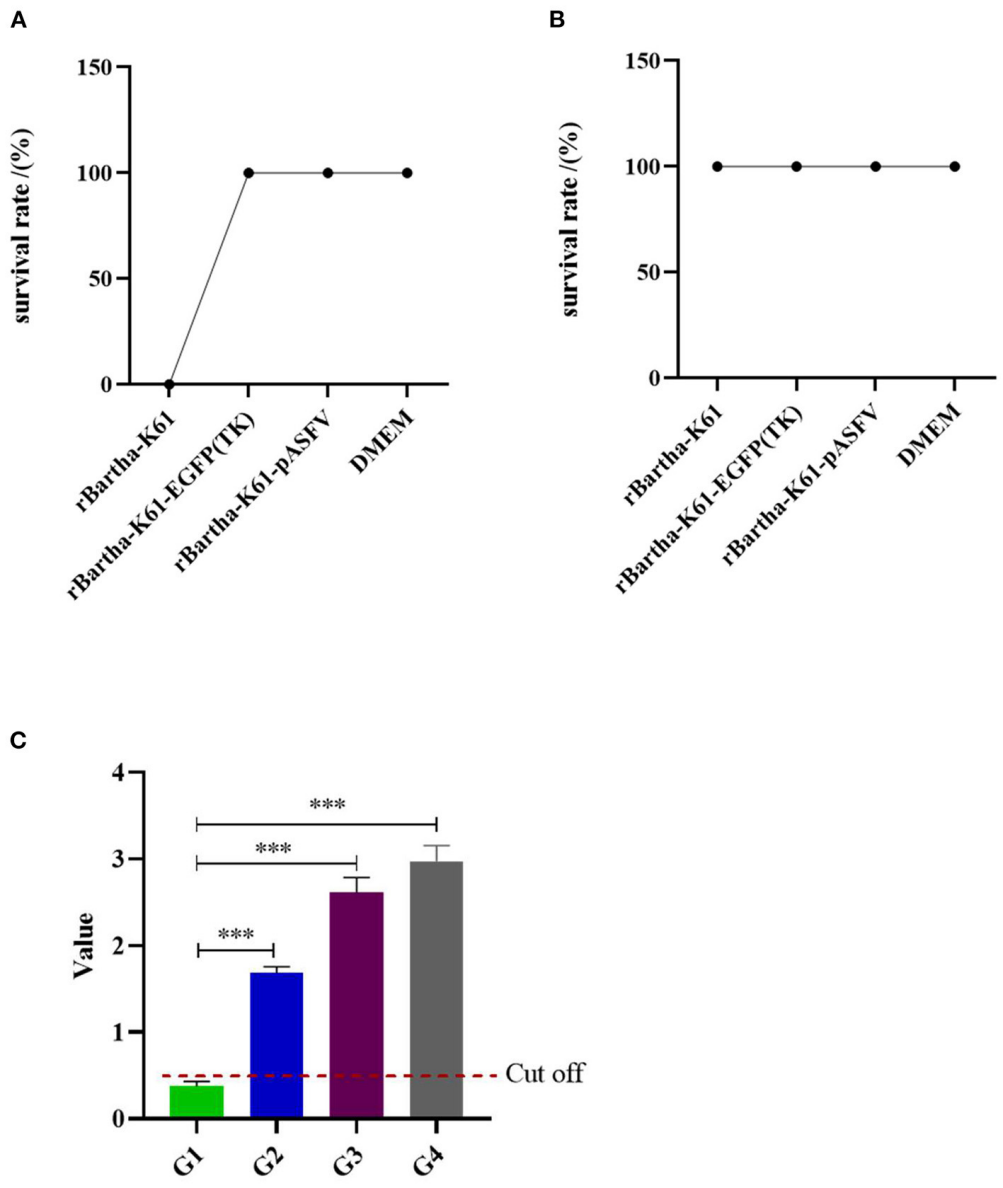
Safety evaluation of recombinant virus in mice and susceptible piglets. **(A)** Safety evaluation of recombinant virus in mice during the 28-day observation period. **(B)** Safety evaluation of recombinant virus in piglets during the 28-day observation period. **(C)** G1–G4 are respectively the mean values of antibodies before inoculation, the average antibody value of 14 days post-inoculation, the average antibody value of 14 days post the second inoculation, the average antibody levels at 28 days post the second inoculation. The horizontal line represents cut-off value (value = 0.510). Bars represent the mean ± SD of over three independent experiments (*n* ≥ 3). Significance was analyzed with the unpaired *t*-test. ***p < 0.001.

To evaluate the safety of the recombinant virus, tests were conducted on mice and susceptible piglets. The details of the sample collection are shown in Figure 4. During the 28-day observation period, rBartha-K61-EGFP (TK), rBartha-K61-pASFV (TK), and DMEM groups showed a healthy state without any symptom reaction. However, on the third day after inoculation, mice in the rBartha-K61 group showed neurological symptoms, such as catch bar and ataxia, and the survival rate dropped to 0 at 4 days after inoculation (Figure 5A), indicating that due to the deletion of the TK gene, the virulence of Bartha-K61 strain was weakened and its pathogenicity to mice was reduced, and the recombinant virus had lower virulence. During the 28-day observation period, all piglets grow healthily without any symptoms (Figure 5B), indicating that the recombinant virus has not changed the virulence of the original vaccine strain Bartha-K61, and the recombinant virus has a good safety for piglets.

After 14 days post-inoculation (dpi), serum of piglets in each group was collected, and antibody levels were detected by ASFV ELISA Kit (Figure 5C). Before inoculation, the antibody p72 was not detected in piglets. After 14 dpi, the antibody level of piglets was significantly increased (*p* < 0.001). In addition, the level of p72 antibody was increased with the increasing inoculation time and inoculation times.

## Discussion

Since August 2018, the worldwide spread of ASF has caused untold economic losses to the pig farming industry (23). The continuous and dynamic situation of the epidemic urgently requires effective prevention and control measures, among which the research and development of new vaccines have an important guiding significance for production. However, due to the long-term co-existence of host and virus, the virus gradually forms various immune escape mechanisms to escape the immune response of the host (2). In addition, the complex and unknown genome of ASFV (24) makes no effective vaccine for ASFV available. Therefore, to do well the prevention and control work of ASF at the present stage, the vaccine research and development work needs to continue.

Currently, various vaccines have been researched and developed, but there is no effective and safe ASFV vaccine (25). The classical inactivated vaccine has good antigenicity, but not enough to resist the attack of ASFV all the immune animals had clinical signs and pathological findings consistent with ASF (26). DNA vaccine can stimulate humoral immunity and cellular immunity, but cannot provide effective protection (27). In this study, the protective antigen of ASFV was inserted into PRV by using Red/ET recombineering technology to obtain a recombinant virus capable of inducing an effective immune response in the body.

The African swine fever virus encodes for at least 150 viral proteins (28), and the main proteins involved in viral replication and the formation of virions include p30 (*CP204L*), pp62 (*CP530R*), p54 (*E183L*), p72 (*B646L*), and CD2v (*EP402R*). A previous report has indicated that pigs immunized with the chimeric protein –p54/30– developed neutralizing antibodies and survived the challenge with a virulent ASFV (29). An ASFV mutant with the deletion of the CD2v and UK was inoculatedpigs, low-level ASFV DNA was detected in blood, nasal swabs, and lymphoid tissue (30). In this study, Red/ET recombineering technology was used to construct recombinant PRV expressing ASFV antigen protein. In the recombinant virus rBartha-K61-pASFV, the ASFV antigen gene successfully replaced the TK gene of the PRV genome, and the recombinant strain could be stably transmitted to 20 generations in PK15 cells, with good stability of gene inheritance and foreign protein expression.

Since the establishment of the reverse genetic system, PRV has been widely used in the study of viral vectors, such as Parvovirus (31), Classical swine fever virus (32), and Foot-and-mouth disease virus (21), with remarkable results. But the virulence of PRV variation strain was significantly enhanced, the infected pigs showed more obvious clinical symptoms and higher mortality, which caused huge economic losses in many pig farms (33, 34). The modified Bartha-K61 vaccine strain with the deletion of non-essential gene TK could significantly reduce the virulence of PRV, but ensure the safety of the vaccine strain and the stable replication of the virus, which made it more widely used in instead of PRV variation strain (35, 36). Therefore, the TK gene was selected as the modification site of the Bartha vector in this study. In the analysis of viral proliferation characteristics, rBartha-K61-pASFV had good proliferation performance, and its proliferation performance on PK15 was similar to the parent virus, with the peak of viral titer up to 1 × 10^5.8^ TCID_50_/ml. But if it can be used in the future, how to improve titer is still to be solved, such as a successful application of the Quality by Design approach to pseudorabies virus production process development in a fixed-bed bioreactor using the serum-free medium (37).

Studies have found that immunization with the cocktail rapidly induced unprecedented ASFV antigen-specific antibody and cellular immune responses against all of the antigens (38). In this study, the recombinant PRV virus expressing ASFV protein was successfully obtained, and the safety was evaluated after being mixed with a cocktail. Based on the diagnostic detection of PRV antibody occurrence in pig farms, combined with the immune program of experimental piglets, we immunize animals with “cocktail” and strengthen the second inoculation two weeks later, in order to rule out the interference of maternal antibodies. The results showed that there were no pruritus, fever, and neurological symptoms in healthy mice and susceptible piglets. The recombinant virus had good safety and could produce p72 antibodies in piglets within a certain time. It is obvious that Bartha-K61-pASFV can stimulate humoral immunity in the body, which provide provides important reference for the screening of antigen genes in ASF vaccine development. But further tests are needed to prove whether Bartha-K61-pASFV can play a protective role against the virus. This is also an urgent problem to be solved in the research and development of ASF vaccine.

In summary, a PRV strain (Bartha-K61) was used as the live virus vector to construct the recombinant virus expressing *CP204L* (p30), *CP530R* (pp62), *E183L* (p54), *B646L* (p72), and *EP402R* (CD2v) genes of ASFV. The recombinant virus strain has stable genetic inheritance and can stimulate the body to produce antibodies.

## Data Availability Statement

The datasets presented in this study can be found in online repositories. The names of the repository/repositories and accession number(s) can be found in the article/supplementary material.

## Ethics Statement

The animal study was reviewed and approved by Committee for Animal Experiments (approval ID: SYXK2019-0136) of South China Agricultural University.

## Author Contributions

LC contributed to the overall experimental design, manuscript writing, and interpretation of all data. ZH, GS, YS, and KF contributed to experimental design and data analysis. ZX contributed to the manuscript revisions. HY contributed to the viral vector construction. HL, WC, WL, XZ, HW, and JF checked and finalized the manuscript. QX contributed to the financial support and experimental design. All authors read and approved the final manuscript.

## Funding

This study was supported by the Key Research and Development Program of Guangdong Province (2020B020222001), the National Key Research and Development Program (Grant No. 2021YFD1801205), the Key-Area Research and Development Program of Guangdong Province (2019B020218004), the Guangdong Basic and Applied Basic Research Foundation (2019A1515012006), and the Construction of Modern Agricultural Science and Technology Innovation Alliance in Guangdong Province (2020KJ128).

## Conflict of Interest

The authors declare that the research was conducted in the absence of any commercial or financial relationships that could be construed as a potential conflict of interest.

## Publisher's Note

All claims expressed in this article are solely those of the authors and do not necessarily represent those of their affiliated organizations, or those of the publisher, the editors and the reviewers. Any product that may be evaluated in this article, or claim that may be made by its manufacturer, is not guaranteed or endorsed by the publisher.
